# Effect of Intraoperative Nerve Monitorization on Predicting Vocal Cord Paralysis in Thyroid Surgery

**DOI:** 10.1055/s-0045-1810079

**Published:** 2026-03-11

**Authors:** Başak Yalçıner, Ömer Bayır, Muhammed Kızılgül, Bekir Uçan, Güleser Saylam, Emel Çadallı Tatar, Erman Çakal, Sibel Alicura Tokgöz, İlker Akyıldız, Mehmet Hakan Korkmaz

**Affiliations:** 1 Department of Otolaryngology and Head and Neck Surgery, Ankara Bilkent City Hospital, Ankara, Turkey; 2 Department of Otolaryngology and Head and Neck Surgery, Etlik City Hospital, Ankara, Turkey; 3 Department of Endocrinology and Metabolism, Etlik City Hospital, Ankara, Turkey; 4 Department of Otolaryngology and Head and Neck Surgery, Lokman Hekim University, Faculty of Medicine, Ankara, Turkey; 5 Department of Otolaryngology, Head and Neck Surgery, Private Practice, Ankara, Turkey

**Keywords:** thyroidectomy, vocal cord paralysis, recurrent laryngeal nerve injuries, intraoperative neurophysiological monitoring, postoperative complications

## Abstract

**Introduction:**

Recurrent laryngeal nerve (RLN) paralysis is a distressing complication of thyroidectomy, and many methods have been investigated for prevention.

**Objective:**

The aim of our study is to investigate to what extent intraoperative neuromonitoring (IONM) predicts postoperative RLN paralysis and what factors may be causing it.

**Methods:**

Patients who underwent thyroidectomy in our clinic between January 2014 and December 2020 were included. Patient diagnoses, demographic characteristics, laboratory and imaging results, surgical information, and IONM results were evaluated.

**Results:**

Data from 287 patients and 523 RLNs were evaluated. Twenty-two (4.2%) of the RLNs were paralytic. The rate of paralysis was significantly higher in total thyroidectomies than in lobectomies ( *p* = 0.046), permanent paralysis rate was higher in left RLNs. Malignancy was statistically associated with permanent paralysis ( *p* = 0.017). The rate of overall and permanent paralysis was higher in patients with negative results of first, second response, first, and second laryngeal twitch examinations. The means of the first and second responses of the paralyzed RLNs were lower ( *p* = 0.016, *p* < 0.01, respectively). The sensitivity of the IONM increased when the new thresholds of the first and second responses were determined to be 155 and 170 µv, respectively, by ROC analysis.

**Conclusions:**

This study demonstrates that voltage variation and magnitude obtained from IONM can be predictive and sensitivity can be increased by using a threshold higher than 100 µV and patients in this range may be at risk for RLN paralysis. Our results may guide future studies of IONM and risk scoring systems to be developed for RLN paralysis.

## Introduction

Recurrent laryngeal nerve (RLN) injury is one of the most important complications of thyroid surgery, a common endocrine procedure. In recent years, with the development of both radiodiagnostic and cytodiagnostic methods, the detection of malignant thyroid disease has increased significantly, leading to an increase in surgical indications. 1 2 3 This situation has led to further investigation of RLN paresis/paralysis, which is one of the most important complications of thyroidectomy. 4 When RLN paralysis is unilateral, it may cause dysphagia, dysphonia, and dyspnea in the patient, and when it is bilateral, the patient may develop upper airway obstruction, which may be fatal. 5 In this surgery, which has been performed for many years, it was previously thought that it was sufficient to visually recognize the RLN and see that its integrity was preserved, but with the development and widespread use of technology, the idea that intraoperative neuromonitoring (IONM) could help prevent or predict this complication has been put forward. IONM provides crucial real-time feedback to surgeons regarding the functionality of the RLN, representing one of the most significant contributions to endocrine surgery in recent decades. 6 Other important factors in RLN monitoring are that several studies have supported that the procedure, when used in a standardized manner, does not add additional comorbidities and that it is also beneficial to the physician from a medicolegal perspective. 6 7

Although IONM is beneficial for nerve mapping and surgeon comfort, especially in cases of difficult thyroidectomy, its effect on the incidence of postoperative vocal cord paralysis is controversial. 8 However, there are studies showing that it is useful in predicting postoperative vocal cord paralysis. 9 Predicting this situation intraoperatively could alert the surgeon to intervene quickly in acute respiratory problems that the patient may experience in the early postoperative period. For this reason, it is important to identify preoperative and intraoperative factors that may cause RLN paralysis and to determine postoperative factors to intervene early in complications that may develop if RLN paralysis persists.

The purpose of this study is to evaluate how well the IONM predicts postoperative temporary or permanent vocal cord paralysis by response to repetitive stimuli and investigating preoperative, intraoperative, and postoperative factors that may be effective in RLN paralysis.

## Methods

This study was conducted in the Department of Otolaryngology and Head and Neck Surgery of our hospital. The authors are accountable for all aspects of the work in ensuring that questions related to the accuracy or integrity of any part of the work are appropriately investigated and resolved. The study was conducted in accordance with the Declaration of Helsinki (as revised in 2013). The study was approved by the institutional ethics committee and individual consent for this retrospective analysis was waived.

This retrospective study was primarily designed to investigate the effect of intraoperative RLN monitoring in predicting postoperative RLN paralysis in patients undergoing total thyroidectomy and thyroid lobectomy. For this purpose, the data of patients who underwent total thyroidectomy and thyroid lobectomy in our clinic between January 2014 and December 2020 were reviewed. Patients who underwent total thyroidectomy/thyroid lobectomy and who underwent intraoperative RLN neuromonitoring for malignant or benign disease in our clinic were included in the study. Patients who underwent central neck dissection/exploration, neck dissection, endoscopic thyroidectomy, laryngectomy + thyroidectomy for cancer invasion, tracheal resection, retrosternal/substernal thyroidectomy, revision cases, patients with preoperative RLN paralysis, Patients whose RLN was sacrificed and could not be identified, cases with non-recurrent RLN, patients with neurological diseases, previous neck surgery, history of neck radiotherapy for head and neck malignancy were excluded from the study.

Gender and age were taken as the demographic data of the patients, and 55 years of age, which is one of the risk criteria for thyroid malignancy, was determined as the age categorization limit. The type of surgery was classified as total thyroidectomy, thyroid lobectomy, and completion thyroid lobectomy. The volume of the largest (dominant) nodule and the volume of the thyroid lobe were calculated according to the Brunn formula based on the preoperative ultrasound measurements of the patients. 10

According to our hospital laboratory's reference ranges, the upper limits of normal were defined as 0.9 IU/ml for anti-thyroglobulin (anti-Tg) and 9 IU/ml for anti-thyroid peroxidase (anti-TPO). Values less than this were considered negative, and values equal to or greater than this were considered positive. The amount of intraoperative bleeding was taken as the amount in milliliters (mL) of the hemorrhagic fluid accumulated in the aspirator after the removal of the washing fluid.

All operations were performed by competent surgeons or under their primary supervision using a Kocher incision. All patients underwent preoperative and postoperative endoscopic examination of the larynx on the first day after surgery. In this way, patients with preoperative paralysis were excluded and those with postoperative RLN paralysis/paresis were detected.

All RLNs were routinely identified macroscopically and surgically dissected and fully exposed. All patients were operated under general anesthesia and intubated with tubes containing IONM-compatible electrodes (Medtronic; Nerve Integrity Monitor (NIM), Jacksonville, FL, USA). In addition, a short-acting neuromuscular blocker was administered to the patient, and it was ensured that no additional doses of neuromuscular blocker were administered to the patient except for intubation. A monopolar stimulation probe was used to stimulate the nerve.

The first response to stimulation when the nerve was first identified was considered the first response, and the response obtained after lobectomy and bleeding control was considered the second response. Stimulation was first applied at a level of 1 mA, and if no response was obtained, 2 mA and then up to 3 mA stimuli were applied. Patients were considered unresponsive if they failed to show an electromyographic response of 100 µV after 3 mA stimulation. The IONM procedure was applied according to the international guidelines published by Randolph et al. in 2010 6 .

In patients who did not respond, the laryngeal twitch response was measured if the probes in the vocal cords did not respond or were dislocated. In this method, which is based on manual assessment of the response of the posterior cricoarytenoid muscle to laryngeal nerve stimulation, the laryngeal nerve, identified visually and tactilely, is stimulated and the movement of the larynx is assessed as present or absent by pressing the posterior part of the larynx with the index finger. The difference between the first and second responses was calculated in µV and the percentage difference between the two responses was noted.

Patients with postoperative paralysis were divided into three groups. Patients with paralysis detected by endoscopy in the first 24 hours after surgery were considered to have RLN paralysis, patients whose RLN paralysis regressed within 6 months were considered to have transient paralysis, and patients who still had RLN paralysis after 6 months were considered to have permanent paralysis.

SPSS 23.0 for Windows program was used for statistical analysis. Normality analysis of continuous variables was evaluated with the Kolmogorov Smirnov test along with descriptive statistics such as skewness and kurtosis. Since the data were not distributed normally in at least one group, all tests were performed non-parametrically. Descriptive statistics: Number and percentage were given for categorical variables, and median (minimum-maximum) for numerical variables. Difference analysis of numerical variables in two independent groups was performed with the Mann Whitney-U test. A comparison of rates in independent groups was made with Chi-Square Analysis. Statistical α significance level was accepted as *p* < 0.05.

## Results

The data of 287 patients who underwent total thyroidectomy/thyroid lobectomy or complementary thyroidectomy and 523 RLNs were evaluated in the study. It was observed that among the patients, 209 (72.8%) were female and 78 (27.2%) were male. Total thyroidectomy was performed in 228 (79.4%) patients while lobectomy was performed in 66 (20.6%) patients. Of the 294 thyroid surgeries performed, paralysis was observed in 22 RLNs (4.2%), of which 11 (2.1%) were temporary and 11 (2.1%) were permanent.

There was no significant difference in the rate of development of permanent or transient paralysis between the sexes.

Statistical analyses showed no association between age and permanent paralysis ( *p* = 0.272). When patients were divided into two categories based on age, namely under and over 55 years of age, based on risk factors related to thyroid cancer, no difference was observed between the two groups in terms of early stage and permanent paralysis.

None of the 67 patients who underwent lobectomy developed vocal cord paralysis. Paralysis was observed in 22 RLNs (4.8%) of patients who underwent total thyroidectomy. Permanent RLN paralysis occurred in 11 RLNs (2.4%) of patients who underwent total thyroidectomy. Although the rate of RLN paralysis was significantly higher in patients who underwent total thyroidectomy ( *p* = 0.046), there was no significant difference between the two groups in the development of permanent RLN paralysis ( *p* = 0.218).

Early paralysis developed in 7 right RLNs (2.7%) and 15 left RLNs (5.6%). There was no significant difference in the development of paralysis between sides ( *p* = 0.101). Permanent paralysis developed in 1 right RLN (0.4%) and 10 left RLNs (3.7%) and the difference was significant ( *p* = 0.008).

Six RLNs (2.8%) of patients with a preoperative diagnosis of benign pathology were observed as paralyzed postoperatively. Seven RLNs (5.1%) of patients with preoperative diagnoses of uncertain significance such as indeterminate atypia and follicular neoplasm were observed as paralytic. Five RLNs (5.2%) of patients operated on with a preoperative diagnosis of hyperthyroidism developed paralysis. Four RLNs (5.8%) of patients with a preoperative diagnosis of malignancy were observed to be paralyzed. There was no statistically significant difference in RLN paralysis between these four groups ( *p* = 0.597).

Two RLNs (0.7%) of patients with a postoperative pathologic diagnosis of benign pathology were observed as paralytic. Nine RLNs (3.7%) of patients with a postoperative pathologic diagnosis of malignancy were observed as paralyzed, and the difference was significant ( *p* = 0.017) ( Table 1 ).

**Table 1:** Patient Characteristics

	n, (%)	
Age (median, min-max)	45 (15–80)	
Sex		
Female	209 (72.8%)	
Male	78 (27.2%)	
Type of Surgery		
Total thyroidectomy	228	
Lobectomy	66	
**Total RLNs**	523 (100%)	
**Paralytic RLNs**	22 (4.2%)	
Transient	11 (2.1%)	
Permanent	11 (2.1%)	
Patients <55 years of age	13 (3.4%)	
Patients ≥55 years of age	9 (6.3%)	
Patients with lobectomy	0 (0%)	
Patients with total thyroidectomy	22 (100%)	
Left RLNs	15 (5.6%)	
Right RLNs	7 (2.7%)	
	**n (%)**	**p value**
**Permanent RLN Paralysis**		0.008 *
Left lobectomy	10 (3.7%)	
Right lobectomy	1 (0.4%)	
Postoperative pathology		0.017 *
Benign	2(0.7%)	
Malignant	9 (3.7%)	
Preoperative Diagnosis		
Benign	5 (2.3%)	0.597 *
Atypia of undetermined significance	3(2.2%)	
Hyperthyroidism	1(1.0%)	
Malign	2(2.7%)	
Lymphocytic Thyroiditis		
Yes	1(0.7%)	0.200 *
No	10(2.6%)	
Antithyroglobulin Antibody		
Positive	0 (0%)	0.268 *
Negative	5 (3.0%)	
Missing	6 (3.6%)	
Antithyroid Peroxidase Antibody		
Positive	1(1.0%)	0.190 *
Negative	7(3.8%)	
Missing	3(2.1%)	
Preoperative lobe volume (mm3, ± SD)		0.400 **
Paralysis	16559.4 ± 8947.1	
No paralysis	18412.2 ± 20814.1	
Preoperative Dominant nodule volume (mm3, ± SD)		0.385 **
Paralysis	3676.8 ± 6198.1	
No paralysis	6619.9 ± 12156.7	

Abbreviations: IQR, interquartile range; RLN, recurrent laryngeal nerve; SD, Standard deviation.

* Chi-Square Test.

** Mann-Whitney-U test.

When the response to neurostimulation was negative at initial identification, paralysis developed in 10 RLNs (12%) and did not develop in 73 RLNs (88%). When the response was positive, paralysis developed in 12 RLNs (2.7%) and did not develop in 428 RLNs (97.3%). The rate of RLN paralysis was significantly higher in patients who had a negative first response ( *p* = 0.001). When the first response to neurostimulation was negative, permanent paralysis developed in 6 RLNs (7.2%) and did not develop in 77 RLNs (92.8%). Permanent paralysis developed in 5 RLNs (1.1%) when the response was positive, while it did not develop in 435 RLNs (98.9%). The rate of permanent RLN paralysis was significantly higher in patients who did not have a positive first response ( *p* = 0.001). After lobectomy and hemorrhage control, 13 RLNs (15.7%) developed paralysis if the response to neurostimulation was negative, while 70 RLNs (84.3%) did not. Paralysis developed in 9 RLNs (2%) when the response was positive, while it did not develop in 432 RLNs (98%). The rate of RLN paralysis was significantly higher in patients who did not receive a second response ( *p* < 0.001). After lobectomy and hemorrhage control, permanent paralysis developed in 8 RLNs (9.6%) if the response to neurostimulation was negative, while it did not develop in 75 RLNs (90.4%). Paralysis developed in 3 RLNs (0.7%) when the response was positive and did not develop in 438 RLNs (99.3%). The rate of permanent RLN paralysis was significantly higher in patients who did not receive the second response ( *p* < 0.001) ( Table 2 ). The sensitivity, specificity, positive predictive value, and negative predictive value of the first and second response for paralysis are also summarized in Table 2 .

**Table 2:** The impact of the first and second responses on all RLN and permanent RLN paralysis, as well as the sensitivity, specificity, positive predictive value, and negative predictive value of the first and second responses

RLN Paralysis				Permanent RLN Paralysis		
	Yes n (%)	No n (%)		Yes n (%)	No n (%)	
			*p value*			*p value*
**First Response**			0,001 *			0,003 *
**Yes**	12 (2.7%)	428 (97.3%)		5 (1.1%)	435 (98.9%)	
**No**	10 (12%)	73 (88%)		6 (7.2%)	77 (92.8%)	
**Second Response**			<0,001 *			<0,001 *
**Yes**	9 (2%)	432 (98%)		3 (0.7%)	438 (99.3%)	
**No**	13 (15.7%)	70 (84.3%)		8 (9.6%)	75 (90.4%)	
**Characteristics of the Test, % (95% CI)**						
	**Sensitivity**		**Specifity**	**Positive Predictive Value**		**Negative Predictive Value**
**First Response- RLN Paralysis**	45.45% (24.39–67.79%)		85.43% (82.03–88.40%)	12.05% (7.64–18.49%)		97.27% (96.05–98.12%)
**Second response- RLN paralysis**	59.09% (36.35–79.29%)		86.23% (82.90–9.12%)	15.85% (11.10–22.13%)		97.96% (96.67–98.76%)
**First response – Permanent Paralysis**	54.55% (23.38- 83.25%)		84.96% (81.57- 87.95%)	7.23% (4.19- 12.19%)		98.86% (97.85–99.40%)
**Second Response- Permanent Paralysis**	72.73% (39.03–93.98%)		85.55% (82.20–88.48%)	9.76% (6.64–14.11%)		99.32% (98.23–99.74%)

* Chi-Square test.

The relationship between a negative result in the first and second laryngeal twitch tests and the development of early and permanent paralysis was found to be significant ( Table 3 ).

**Table 3:** The findings of the first and second laryngeal twitch examinations performed on patients who did not receive the first response, and the relationship between early-stage and permanent paralysis, as well as the sensitivity, specificity, positive predictive value, and negative predictive value of this test

**RLN Paralysis**	**n(%)**			
	**Yes (** ***n*** ** = 22)**	**No(** ***n*** ** = 501)**	**p value**	
**Laryngeal Twitch-1**			**<0.001** *	
**Yes**	2(3.2%)	60(96.3%)		
**No**	8(38.0%)	13(62.0%)		
**Permanent RLS Paralysis**				
	**Yes (** ***n*** ** = 11)**	**No (** ***n*** ** = 512)**		
**Laryngeal Twitch-1**			**0.001** *	
**Yes**	1(1.6%)	61(98.4%)		
**No**	5(2.4%)	16(97.6%)		
**RLN Paralysis**				
	**Yes (** ***n*** ** = 22)**	**No(** ***n*** ** = 501)**		
**Laryngeal Twitch-2**				
**Yes**	3(4.8%)	59(95.2%)	**<0.001** *	
**No**	10(45.4%)	11(54.6%)		
**Permanent RLS Paralysis**				
	**Yes (** ***n*** ** = 11)**	**No (** ***n*** ** = 512)**		
**Laryngeal Twitch-2**				
**Yes**	2 (3.2%)	60 (96.8%)	**0.001** *	
**No**	6 (28.6%)	15 (74.1%)		
**Characteristics of the Test, % (95% CI)**				
	**Sensitivity**	**Specificity**	**Positive Predictive Value**	**Negative Predictive Value**
**Laryngeal Twitch 1- RLN Paralysis**	80.00% (44.39–97.48%)	82,.9% (71.47–90.16%)	38.10% (25.58–52.41%)	96.77% (89.63–99.05%)
**Laryngeal Twitch 1- Permanent RLN Paralysis**	83.33% (35.88–99.58%)	79.22% (68.46–87.63%)	23.81% (15.09–35.46%)	98.39% (91.04–99.73%)
**Laryngeal Twitch 2- RLN Paralysis**	76.92% 46.19–94.96%)	84.29% (73.62–91.89%)	47.62% (32.87–62.80%)	95.6% (32.87–62.80%)
**Laryngeal Twitch 2- Permanent RLN Paralysis**	75.00% (34.91–96.81%)	80.00% (69.17–88.35%)	28.57% (17.94–42.26%)	96.77% (89.99–99.01%)

* Chi-Square test.

**Fig. 1:**
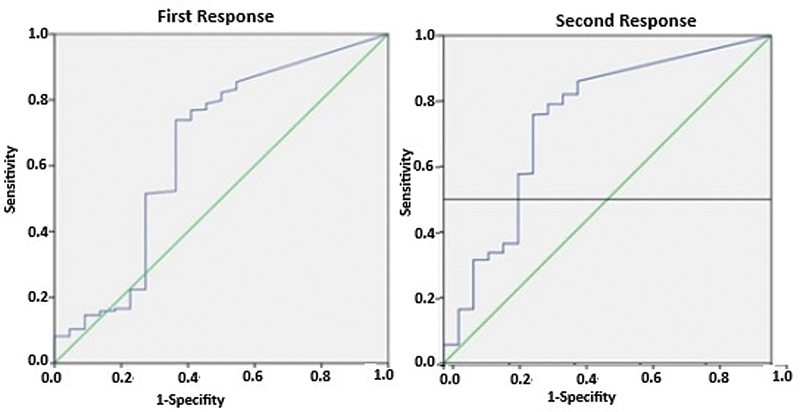
ROC analysis curve showing the relationship between the first and second responses and RLN paralysis.

The mean response obtained with 1 mA neurostimulation at the time of initial identification during surgery was 277 +/− 370.7 µV for the 22 RLNs that were paralytic, whereas it was 423 +/− 420.9 µV for the 501 RLNs that were not paralytic. The responses of the paralytic RLNs are significantly lower ( *p* = 0.016). ROC analysis identified a threshold of 155 µV for the first response (AUC: 0.651, *p* = 0.017) ( Fig. 1 ). The mean response obtained by 1 mA neurostimulation after lobectomy and hemorrhage control was 208.7 +/− 341.7 µV for the 22 paralytic RLNs, while it was 492.9 +/− 444.9 µV for the 501 non-paralytic RLNs. The second responses of the paralytic RLNs was significantly lower ( *p* < 0.001). The ROC analysis determined a cut-off value of 170 µV for the second response (AUC: 0.740, *p* < 0.001) ( Fig. 1 ).

According to the new threshold value determined in our study, the rate of RLN paralysis was significantly higher in patients who did not receive the first and second responses ( Table 4 ).

**Table 4:** The relationship between the first and second responses with early-stage and permanent paralysis based on the new threshold value, and the sensitivity, specificity, positive predictive value, and negative predictive value of the new threshold values

**RLN Paralysis**	**n (%)**			
	**Yes (** ***n*** ** = 22)**	**No(** ***n*** ** = 501)**	***p*** *	
**First Response**			**<0.001**	
**Yes**	8(2.1%)	373 (97.9%)		
**No**	14(9.9%)	128 (90.1%)		
**Permanent RLS Paralysis**				
	**Yes (** ***n*** ** = 11)**	**No (** ***n*** ** = 512)**		
**First Response**			**0.001**	
**Yes**	3 (0.8%)	375 (99.2%)		
**No**	8 (5.5%)	137 (94.5%)		
**RLN Paralysis**				
	**Yes (** ***n*** ** = 22)**	**No(** ***n*** ** = 501)**		
**Second Response**				
**Yes**	6 (1.5%)	382 (98.5%)	**<0.001**	
**No**	16(11.9%)	119 (88.1%)		
**Permanent RLS Paralysis**				
	**Yes (** ***n*** ** = 11)**	**No (** ***n*** ** = 512)**		
**Second Response**				
**Yes**	3 (0.8%)	383 (99.2%)	**0.001**	
**No**	8 (5.8%)	129 (94.2%)		
**Characteristics of the Test, % (95% CI)**				
	**Sensitivity**	**Specificity**	**Positive Predictive Value**	**Negative Predictive Value**
**First Response - RLN Paralysis**	63.64% (40.66–82.80%)	74.45% (70.39–78.22%)	9.86% (7.16–13.43%)	94.40% (94.40–98.78%)
**First Response Permanent RLN Paralysis**	72.73% (39.03–93.98%)	73.24% (69.18–77.03%)	5.52% (3.81–7.93%)	99.21% (97.94–99.70%)
**Second Response- RLN Paralysis**	72.73% (49.78–89.27%)	76.25% (72.27–79.91%)	11.85% (9.06–15.36%)	98.45% (96.98–99.41%)
**Second Response -Permanent RLN Paralysis**	72.73% (39.03–93.98%)	74.80% (70.81–78.51%)	5.84% (4.02–8.40%)	99.22% (97.98–99.70%)

* Chi-Square test.

Additionally, we analyzed the relationship between the second response and paralysis in 83 patients who did not have a first response. Transient paralysis was observed in 4 (4.8%) of these patients. None of these four patients had a second response. Transient paralysis did not develop in 68 patients (81.9%) who had no second response. None of the 11 (13.3%) patients who had a second response experienced transient paralysis ( *p* = 0.42). Permanent paralysis developed in 6 patients (7.2%) without a second response, while it did not develop in 66 patients (79.5%). None of the 11 patients (13.3%) who had a second response developed permanent paralysis ( *p* = 0.32).

The 440 patients who had a first response were also evaluated separately. For transient paralysis, the test had a sensitivity of 14.29%, specificity of 97.69%, positive predictive value of 9.09%, and negative predictive value of 98.60% ( *p* = 0.04). For permanent RLN paralysis, the sensitivity was 40%, specificity 97.93%, positive predictive value 18.18%, and negative predictive value 99.30% ( *p* = 0.001). The findings are summarized in Table 5 .

**Table 5:** The impact of second responses on transient and permanent RLN paralysis, as well as the sensitivity, specificity, positive predictive value, and negative predictive value of the second responses in patients with a positive first response

Transient Paralysis				Permanent RLN Paralysis		
	Yes n (%)	No n (%)		Yes n (%)	No n (%)	
			*p value*			*p value*
**Second Response**			0.04 *			0.001 *
**Yes**	6 (9.1)	423 (90.9%)		3 (0.7%)	426(99.3%)	
**No**	1 (9.1%)	10 (90.9%)		2 (18.2%)	9 (81.8%)	
**Characteristics of the Test, % (95% CI)**						
	**Sensitivity**	**Specificity**	**Positive Predictive Value**	**Negative Predictive Value**		
**Second response- Transient RLN paralysis**	14.29% (0.36–57.87%)	97.69% (95.79–98.89%)	9.09% (1.45–40.44%)	98.60% (98.12–98.96%)		
**Second Response- Permanent Paralysis**	40.00% (5.27–85.34%)	97.93% (96.11–99.05%)	18.18% (5.97–43.76%)	99.30% (98.58–99.66%)		

* Chi-Square test.

## Discussion

RLN paralysis is one of the most feared complications of thyroidectomy, and many studies have been conducted and methods have been investigated to prevent and predict it. Intraoperative RLN monitoring helps to identify the nerve and prevent its paralysis, especially in high-risk cases, and our study aims to contribute to the investigation of the benefits of this method and the prediction of RLN paralysis. The strengths of our study, which included 287 patients and 523 RLNs, are the large sample size, the long follow-up period, and the fact that it was designed to study early, temporary, and permanent paralysis separately.

IONM is now a widely used method in thyroid surgery, and the threshold for the presence of a response is widely accepted as 100 µV. 6 11 However, there are very few studies in the literature on the numerical value of muscle response to intraoperative RLN stimulation, and the prognostic significance of changes in this value is unknown.

The retrospective nature of the study and the fact that only thyroidectomy and lobectomy patients were included may be considered limitations of the study. However, difficult thyroidectomy cases such as central neck dissection, revision thyroidectomy, and thyroidectomy combined with parathyroid surgery, which are risk factors for RLN paralysis, were not included in the study to create a homogeneous patient population. In view of the results of this study, it may be considered to evaluate these groups in future studies. Additionally, a potential limitation of our study is the equal distribution of permanent and transient RLN paralysis (1:1 ratio), which differs from the typical 3–4:1 ratio reported in the literature. 12 13 While our permanent paralysis rate (2.1%) aligns with accepted standards, our transient paralysis rate appears lower than usually reported, possibly due to our institution being a tertiary referral center receiving more complex cases, our higher proportion of malignant cases, which is considered a risk factor for permanent RLN paralysis, 14 15 and our rigorous endoscopic follow-up protocol.

In our study, permanent paralysis was observed to be significantly higher on the left side, and although similar results have not been observed in the literature, the reasons may be that more careful dissection is required due to the greater variation and complexity of the anatomy on the right side, and that extralaryngeal branching, which is a risk factor for nerve injury, is more common on the left side. 16

There are conflicting data in the literature regarding the relationship between preoperative and postoperative disease diagnosis and RLN paralysis. The study by Moreira et al. published in 2020, including 1003 patients, reported that preoperative malignancy was not associated with RLN paralysis. 13 In a retrospective study of 14,934 patients in 2004, the rate of RLN damage in patients operated on with a diagnosis of thyroid cancer was reported to be 5.7%, and a significant difference was found between cancer type and paralysis rate. 17 Another study reported that cancer surgery was a risk factor for RLN damage, but noted that the cause of the risk was often due to invasion of the nerve and surrounding soft tissue. 18 The reason for the lack of difference in malignant diseases in our study may be that patients with vocal cord paralysis on preoperative endoscopic laryngeal examination and intraoperative macroscopic tumor invasion in and around the laryngeal nerve were excluded. Apart from this, the higher rate of permanent paralysis in patients with a postoperative pathological diagnosis of malignancy may be explained by the fact that the tissues are fibrotic and dissection is more difficult, which is consistent with the general literature. 18 19

Thyroidectomy for hyperthyroidism is known to be more difficult than for other etiologies, mainly due to increased vascularity. 20 21 In addition, the study by Mok et al. found that complication rates were significantly higher in difficult thyroidectomies. 21 Therefore, we included patients who underwent thyroidectomy for hyperthyroidism in our study. Although there are studies reporting high complication rates when evaluated in terms of RLN paralysis, some studies did not find a significant association between any benign etiology, including Graves' disease, and the increased risk of RLN paralysis. 18 22 23 24 25 However, one study suggested that anti-TG positivity was associated with difficult thyroidectomy. 21 A study published in 2012 by McManus et al. showed that Hashimoto's thyroiditis was associated with an increased complication rate, 26 while the study by Moreira et al. reported that there was no significant association between Hashimoto's thyroiditis and RLN paralysi. 14 When these studies are evaluated, it seems that lymphocytic thyroiditis and thyroid autoimmunity are risk factors for difficult thyroidectomy, but it is controversial regarding the development of RLN paralysis. In our study, no significant association was found between the pathology of lymphocytic thyroiditis and thyroid autoantibodies and RLN paralysis. In addition, the rate of RLN paralysis in patients who underwent surgery for hyperthyroidism was not different from that in other patients.

In a retrospective study of one hundred and forty-six patients, a significant association between thyroid lobe size and temporary paralysis was reported. 27 However, in the same study, no significant association was found for permanent paralysis. The reasons for the differences in our findings may be the exclusion of revision and retrosternal goiter cases in our study, the amount of bleeding is significantly higher in cases with temporary paralysis, and differences in imaging techniques.

Although there are no studies in the literature on the dominant nodule size in terms of its effect on difficult thyroidectomy and RLN paralysis, a study published by Kwak et al. in 2015, including 1293 patients, reported that the dominant nodule size calculated by preoperative ultrasound was not associated with prolonged surgery. 28 In our study, there was no significant relationship between dominant nodule size and RLN paralysis.

The routine use of IONM and its role in preventing RLN damage in primary thyroid surgery is controversial. A systematic review of five randomized controlled trials concluded that there was insufficient data to recommend IONM for all patients undergoing thyroid surgery or for patients at high risk of RLN paralysis. 29 Another systematic review conducted by Wong et al in 2016, which included 10 different studies, found that the use of IONM in revision cases and thyroidectomies performed for malignancy reduced the overall rate of RLN paralysis. 8

Today, visual identification of the RLN is still considered the gold standard in preventing paralysis. 23 The 2020 guideline of the American Association of Endocrine Surgeons emphasized that none of the studies on the use of IONM showed significant data in preventing RLN paralysis and stated that its use is common in selected cases (such as reoperation, large or substernal goiter, locally advanced thyroid cancer). 30

Additional analyses showed that none of the patients with a second response who lacked a first response developed paralysis. However, the percentage of patients without a response who did not develop transient paralysis was also high at 81.9%. Although this did not reach statistical significance, likely due to the small number of patients, this finding suggests a high negative predictive value of the second response in patients without a first response, though verification with larger cohorts is necessary. In our additional analysis evaluating the second response in patients who had a first response, despite the test's high specificity and negative predictive value, its low sensitivity value limits its use for screening purposes. While the second response may have higher diagnostic value, particularly for permanent RLN paralysis, these results need to be confirmed in larger patient populations. In addition, it should be kept in mind that although the specificity of the test is consistently reported to be high, positive predictive values vary considerably, ranging from 30–80%, indicating that positive results (i.e., no response) should be carefully interpreted. 6 31

In our study, we found that IONM had a positive predictive value of 9.09–18.18% for RLN paralysis, which is lower than some previously reported values in the literature. For instance, a comparable prospective study examining 1000 nerves at risk reported a much higher positive predictive value of 76.7% using the standard 100μV threshold, and 77.4% when using their optimized threshold of 189μV. 32 This discrepancy could be attributed to several factors, including differences in patient populations, surgical techniques, or monitoring equipment. Interestingly, our ROC analysis suggested optimal thresholds of 155μV for the first response and 170μV for the second response, which is relatively close to the finding of 189μV as an optimal cutoff. In alignment with a comprehensive systematic review examining 44,575 nerves at risk, our findings confirm that while IONM demonstrates consistently high negative predictive values, its relatively low positive predictive value limits its use as a standalone predictive tool for RLN paralysis—a limitation that may be partially addressed by adopting optimized voltage thresholds rather than relying on the conventional 100μV cutoff. 33 Despite these varying results across studies, the consistently high negative predictive values across all studies, including ours, confirm that IONM remains a valuable tool for predicting normal postoperative nerve function, particularly in high-risk cases.

We believe that it is useful in mapping the nerve, guiding its dissection, and estimating the location of the nerve lesion, especially in high-risk procedures such as Graves' surgery, large goiter, revision cases, and cases of variation such as extralaryngeal branching of the RLN and non-recurrence. Consistent with the literature, the negative predictive value of IONM in our study was high. 8 34 For the above reasons, we believe that the main use of IONM is to provide additional benefit in ruling out RLN damage in difficult surgical cases, since lack of response may be due to many reasons other than RLN damage.

Our study showed that there was a significant relationship between the lack of first and second response and the development of early and permanent RLN paralysis. There was a significant relationship between lack of response and RLN paralysis in the laryngeal twitch test performed in patients with no first or second response. It was observed that the negative predictive value of the laryngeal twitch test was similar to that of the IONM responses, which is also consistent with the literature. 34 In addition, it was observed that the sensitivity of the test increased with the newly determined thresholds. However, to increase the reliability of this test, which has a very high negative predictive value, it should be noted that temporary and permanent RLN paralysis was observed in patients who responded above 100 µV but below the specified thresholds. Although the negative predictive value is quite high, it should not be ignored that the nerve responding to the stimulus may also be paralyzed. As mentioned above, although the threshold of 100 µV for the presence of a response is widely accepted, to our knowledge, our study is the first to compare the voltage difference and the numerical value of the voltage.

## Conclusions

RLN paralysis is an important complication of thyroid surgery, and despite the development of IONM methods, visual identification of the nerve is still the gold standard for preventing paralysis. There is no standardized prognostic model, but many risk factors were evaluated in this study to predict RLN paralysis. In addition, this study shows that voltage changes and its numerical value during IONM can be predictive. It was shown that sensitivity can be increased by using thresholds higher than the commonly accepted value of 100 µV. This finding suggests that rather than using a single threshold, it may be appropriate to evaluate patients within a range of values for RLN paralysis. However, it should be kept in mind that IONM is a supportive test, and technical errors may occur for the reasons mentioned above, and it would not be appropriate to use it instead of visual identification of the nerve. This study may guide future prospective studies of IONM and risk-scoring systems that may be developed for RLN palsies.

## Highlights

- Prediction tools for vocal cord paralysis, a dreaded complication in thyroid surgery, remain an unmet need.
- IONM effectively predicted both temporary and permanent vocal cord paralysis.
- Using higher voltage thresholds may be reasonable in high-risk patients for increasing sensitivity.
- More extensive surgery, site, and malignancy were identified as high-risk factors.
- Validation of these results in larger cohorts may lead to the development of nomograms with the integrating of other clinical risk factors for improved risk assessment.
